# Supplementary material for: A Silicone Resin Coating with Water-Repellency and Anti-Fouling Properties for Wood Protection
Source: Polymers (Basel). 2022 Jul 28;14(15):3062. doi: 10.3390/polym14153062 (PMC9370440; doi:10.3390/polym14153062)
Supplement: Supplementary file 1 [file polymers-14-03062-s001.zip › polymers-1807214-Supplementary materials.pdf]

Supplementary Materials

Zehao Ding, Wensheng Lin, Wenbin Yang, Hanxian Chen * and Xinxiang Zhang *

College of Materials Engineering, Fujian Agriculture and Forestry University, Fuzhou 350108, China; 15396095156@163.com (Z.D.); wensheng0817@163.com (W.L.); fafuywb@163.com (W.Y.)

* Correspondence: hanxian1229@163.com (H.C.); xxzhang0106@163.com (X.Z.)

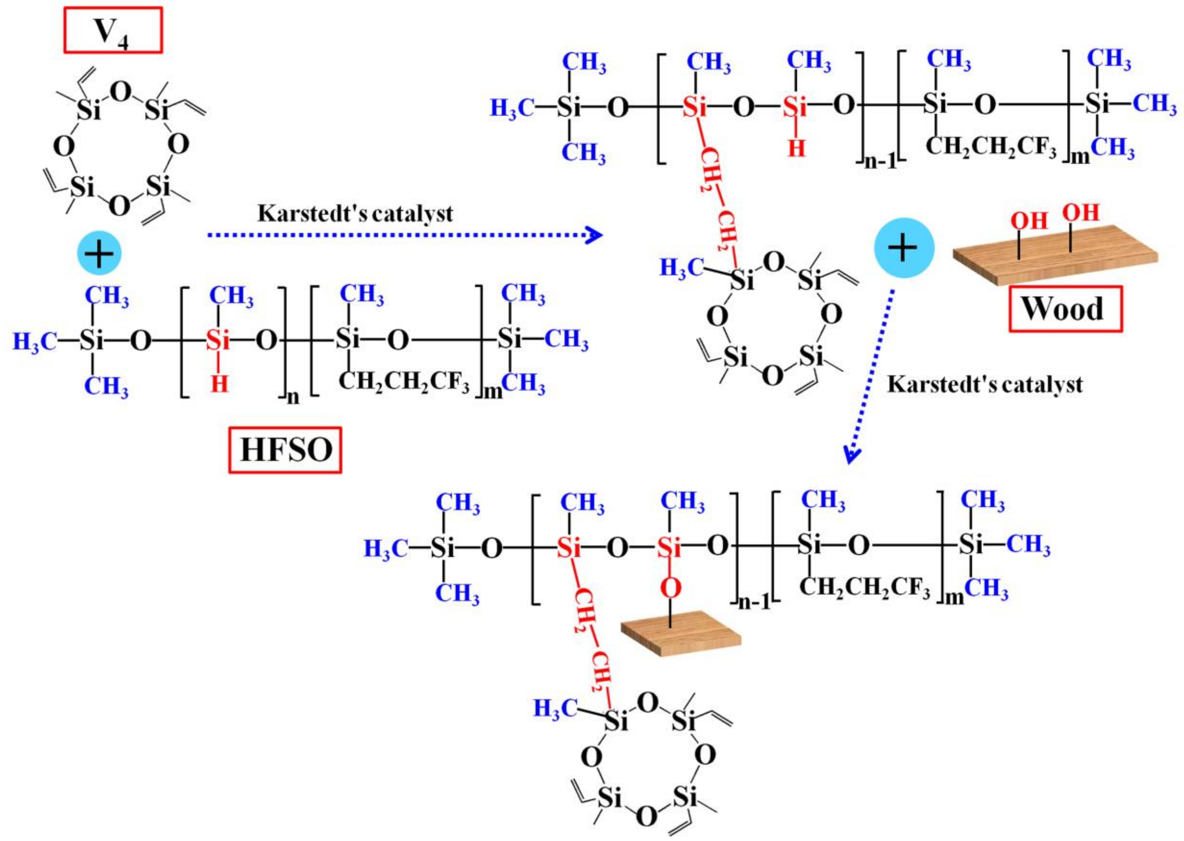

Figure S1: Schematic diagram of grafting silicone resin on wood surface

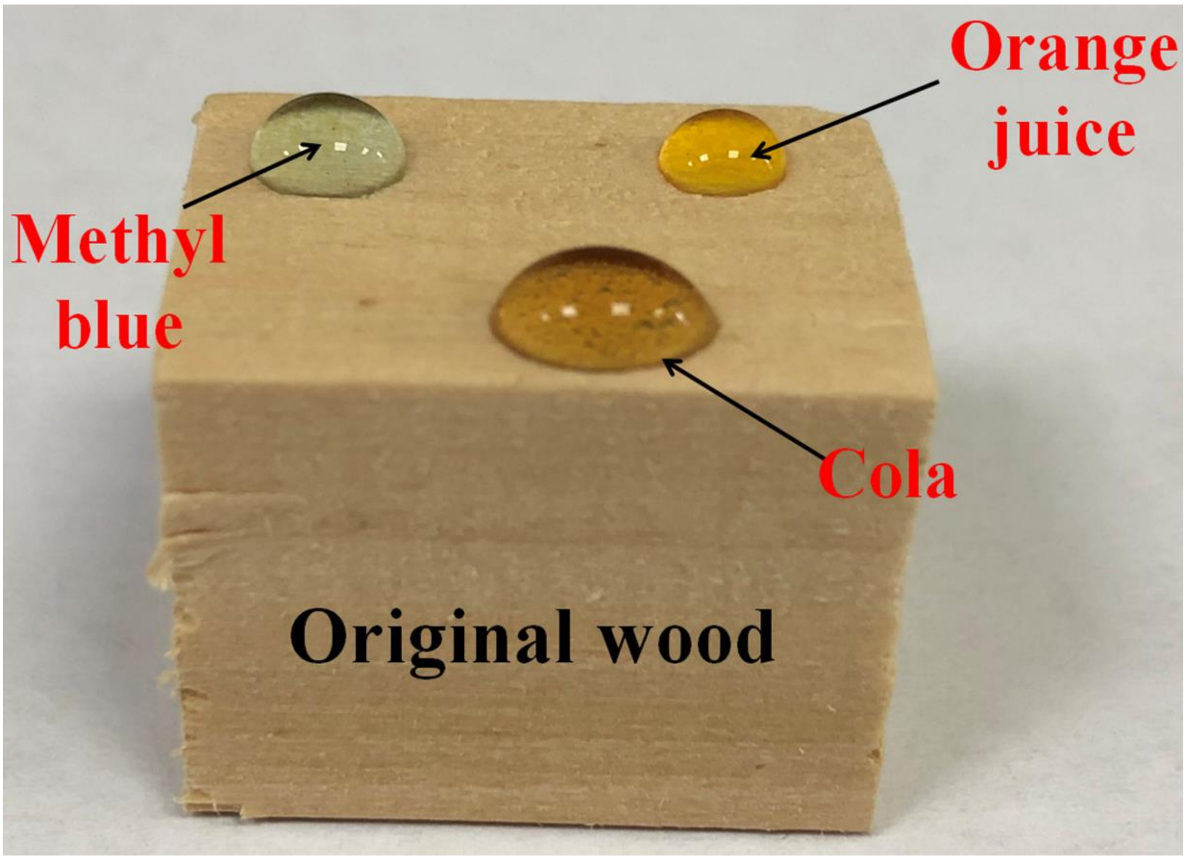

Figure S2: Anti-foulig resistance of unmodified wood
